# Multidisciplinary Management of Opioid Use–Related Infective Endocarditis: Treatment, QTc Values, and Cardiac Arrests due to Ventricular Fibrillation

**DOI:** 10.3390/jcm12030882

**Published:** 2023-01-22

**Authors:** Lynda E. Rosenfeld, Shashank Jain, Andrea Amabile, Arnar Geirsson, Markus Krane, Melissa B. Weimer

**Affiliations:** 1 Section of Cardiovascular Medicine, Yale School of Medicine, New Haven, CT 06510, USA; 2 Section of Cardiovascular Medicine, Case Western Reserve School of Medicine, Cleveland, OH 44106, USA; 3 Division of Cardiac Surgery, Yale School of Medicine, New Haven, CT 06510, USA; 4 Division of General Medicine, Program in Addiction Medicine, Yale School of Medicine, New Haven, CT 06510, USA

**Keywords:** opioid use disorder, infective endocarditis, methadone

## Abstract

(1) Background: The opioid epidemic has led to an increase in cardiac surgery for infective endocarditis (IE-CS) related to injection use of opioids (OUD) and other substances and a call for a coordinated approach to initiate substance use disorder treatment, including medication for OUD (MOUD), during IE-CS hospitalizations. We sought to determine the effects of the initiation of a multi-disciplinary endocarditis evaluation team (MEET) on MOUD use, electrocardiographic QTc measurements and cardiac arrests due to ventricular fibrillation (VF) in patients with OUD. (2) Methods and Results: A historical group undergoing IE-CS at Yale-New Haven Hospital prior to MEET initiation, Group I (43 episodes of IE-CS, 38 patients) was compared to 24 patients undergoing IE-CS after MEET involvement (Group II). Compared to Group l, Group II patients were more likely to receive MOUD (41.9 vs. 95.8%, *p* < 0.0001), predominantly methadone (41.9 vs. 79.2%, *p* = 0.0035) at discharge. Both groups had similar QTcs: approximately 30% of reviewed electrocardiograms had QTcs ≥ 470 ms and 17%, QTcs ≥ 500 ms. Cardiac arrests due to VF were not uncommon: Group I: 9.3% vs. Group II: 8.3%, *p* = 0.8914. Half occurred in the 1–2 months after surgery and were contributed to by pacemaker malfunction/ management and half were related to opioid use. (3) Conclusions: MEET was associated with increased MOUD (predominantly methadone) use during IE-CS hospitalizations without an increase in QTc prolongation or cardiac arrest due to VF compared to Group I, but events occurred in both groups. These arrests were associated with pacemaker issues or a return to opioid use. Robust follow-up of IE-CS patients is essential, as is further research to clarify the longer-term effects of MEET on outcomes.

## 1. Introduction

The ongoing increase in the use of injection drugs, including opioids, in the United States has been associated with an epidemic not only of unintentional overdoses [1] but of infectious complications in general and infective endocarditis (IE) in particular. Among all hospitalizations for IE, those associated with injection, opioid use and opioid use disorder (OUD) increased from 6.3% in 2005 to 11.6% in 2014 [2], leading to a call to action by the American College of Physicians, the Infectious Diseases Society of America and the National Institutes of Health [3]. Part of this call involved a recommendation to create hospital-based protocols to facilitate initiation of medication treatment for OUD (MOUD) during the IE hospitalization, to coordinate care among disciplines including cardiology, cardiothoracic surgery, infectious diseases, addiction medicine, addiction psychiatry and social work, and to support patient transitions to outpatient addiction treatment programs on hospital discharge [4]. MOUD, including methadone and buprenorphine, can be safely and effectively initiated in the hospital setting. While methadone is a full opioid agonist, buprenorphine is a partial opioid agonist, and their differences may influence which agent is chosen for treatment of IE patients undergoing cardiac surgery (IE-CS) [5]. For those newly initiating MOUD in the setting of IE-CS, methadone may be a more ideal choice to quickly treat opioid withdrawal and pain peri-operatively. However, methadone, in contrast to buprenorphine, has been associated with electrocardiographic QT prolongation and torsades de pointes [6]. From 10 to 56% of individuals on long-term methadone treatment for OUD develop moderate QT prolongation [7,8,9,10] and approximately 2% [7,8,9,10] develop a QTc > 500 ms, placing them at the highest risk for torsades de pointes/VF. The inflammation, stress, electrolyte abnormalities and polypharmacy associated with IE-CS may heighten these risks. Thus, the choice of one medication over another should be a shared decision between clinicians, ideally including addiction medicine specialists, and the patient. To address the care complexity of individuals with IE-CS and with the goal of ameliorating outcomes, a multi-disciplinary endocarditis evaluation team (MEET) [4] was developed at Yale-New Haven Hospital (YNHH), an urban tertiary care hospital. We, therefore, sought to examine the use of MOUD and methadone in particular during hospitalizations for IE-CS, its effect on the QTc, and the risk of cardiac arrest due to ventricular fibrillation (VF)/torsades de pointes during and after hospitalization for IE-CS in patients with OUD, just before and immediately after the initiation of a MEET at YNHH.

## 2. Materials and Methods

We performed a retrospective cohort study of people with OUD undergoing IE-CS at YNHH who survived surgery and for whom follow-up was available for at least 1 month post-IE-CS. Patients treated immediately before (8/2013–11/2018) (Group I: 43 episodes of IE-CS, 38 unique patients) and just after (11/2018–1/2020) (Group II: 24 episodes of IE-CS, 24 unique patients) initiation of the MEET were compared. This team, comprised of addiction medicine, anesthesia, cardiology, cardiac surgery and infectious disease clinicians, as well as case managers, nurses and social workers, was designed to optimize, formalize and standardize care for patients with OUD and IE [4]. While some Group I patients (22) may have been seen casually by addiction medicine, psychiatry, pain management or palliative care clinicians, none received longitudinal or coordinated follow-up by any of these services and often had only single visits to deal with an acute issue.

Our specific goal was to determine if the use of MOUD in the 2 cohorts was different and if this affected electrocardiographic findings, specifically QTc prolongation, and cardiac arrest due to VF.

Patients were defined as having endocarditis based on the Center for Disease Control and Prevention surveillance definition [11]. This definition has been adopted by the Society of Thoracic Surgeons (STS) Adult Cardiac Surgery Database and is similar to the Duke Criteria for IE. In both groups, OUD was defined based on the STS Adult Cardiac Surgery Database specifications, indicating whether there was a documented history of injection opioid use. Group II patients also had a formal diagnosis of OUD based on the American Psychiatric Association Diagnostic and Statistical Manual of Mental Disorders (DSM-5) [12] as assessed by medically trained addiction physicians. This study was approved by the Yale Institutional Review Board, and individual patient consent was waived.

Data were collected from the YNHH electronic health record and linked to the institutional STS database.

Twelve lead electrocardiograms obtained as part of routine clinical care were examined at 3 space: just after admission, shortly after IE-CS and peri-discharge. These were reviewed by an electrophysiologist. QT calculations were conducted with electronic calipers using the tangent method in Lead II, if at all possible. If a distinct T wave was not apparent in Lead II, the lead with the clearest T wave was used. Bazett’s formula was used to calculate the corrected QT (QTc). In patients with a prolonged QRS (either a paced QRS or intraventricular conduction defect) a “corrected” QTc (cQTc) was calculated using the formula: cQTc = QT-QRS/2 /^2^$\sqrt{RR}$ and was used in preference to the QTc in all appropriate patients [13]. Adjustments in MOUD were created according to routine care by managing clinicians. Cardiac arrests were defined as clinical events with documented ventricular fibrillation or torsades de pointes, based on chart review.

Normally distributed, continuous variables were reported as mean ± standard deviations; non-normally distributed, continuous variables were reported as median and interquartile ranges. Categorical variables were reported as absolute and relative frequencies. Student’s *t* test, Wilcoxon rank-sum test, and ANOVA test were used to compare mean and median values, as deemed appropriate; chi-squared test and Fisher’s exact test were used to compare proportions. Statistical analysis was performed using RStudio 1.3.1073 (RStudio, PBC). A two-sided *p*-value of < 0.05 was the criterion for statistical significance.

## 3. Results

The patients in both groups were relatively young (Group I: 37.0 ± 12.5 (mean ± SD): years old; Group II: 38.1 ± 11.4 years old). The majority had been infected with the Hepatitis C virus, as determined by a positive blood test for Hepatitis C antibodies (Group I: 68.4% vs. Group II: 66.7%, *p* = 0.890) and those in Group I were less likely to have left ventricular dysfunction (left ventricular ejection fraction < 50%) (2.6% vs. 25%, *p* = 0.007). Only a minority had HIV infection (Table 1). As might be anticipated in a population with OUD-associated IE, the tricuspid valve was most commonly involved (32/79 infected valves). The aortic (24/79) and mitral (23/79) valves were infected less often. By far the most common causative organism was staphylococcus (37/69 organisms identified), followed by streptococcus (17/69) and fungal infections (9/69).

Compared to Group I, MOUD was more likely to be initiated, used and maintained during Group II IE-CS episodes (41.9% vs. 95.8%, *p* < 0.0001). Buprenorphine was not prescribed at discharge for any Group I patient but was prescribed for four Group II patients (*p* = 0.014). Patients in Group II were more likely to be receiving methadone early in their hospitalization (although it may have continued as previously prescribed therapy, it was newly initiated on admission in 1 of 16 patients in Group I but in 6 of 18 patients in Group II), after surgery and at discharge, and almost twice as many patients with input from MEET were discharged receiving methadone than in Group I (47.4% vs. 79.2%, *p* = 0.0134 patients, 41.9% vs. 79.2% IE-CS episodes, *p* = 0.0035 (Table 1)).

In patients receiving methadone, median doses in both groups were similar throughout the period of hospitalization (Table 2).

**Figure 1:**
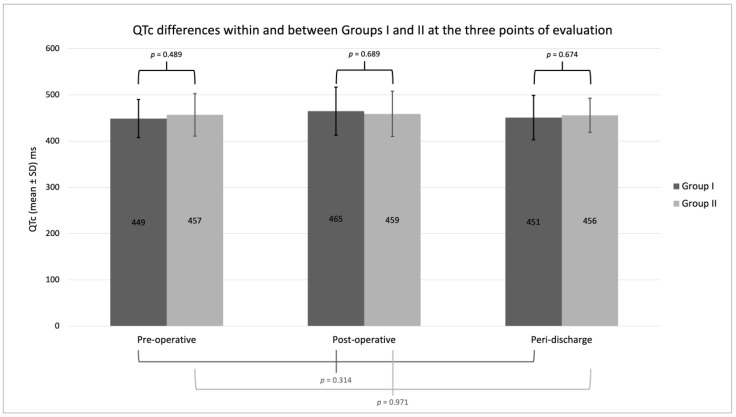
QTc differences within and between Groups I and II at the three points of evaluation (pre-surgery, post-surgery and peri-discharge). ms = milliseconds; QTc = corrected QT (includes QTc corrected for prolonged QRS, cQTc, where appropriate); SD = standard deviation.

Both groups were exposed to many QT-prolonging factors in addition to MOUD during their hospitalizations. These included other medications (antibiotics, antifungals, antiemetics, antiarrhythmics, etc.) and electrolyte abnormalities. This did not differ significantly between the groups and peaked at approximately four QT-prolonging factors at the time of surgery. Patients in both groups had moderately prolonged QTcs. Overall, 33% of Group I, and 30% of Group II electrocardiograms that were reviewed had a QTc or cQTc ≥ 470 ms and 17% of both Group I and II patients had at least one QTc or cQTc ≥ 500 ms despite the more frequent use of methadone in Group II patients. These values did not differ significantly over time in either group or between groups at the three time points surveyed (Figure 1).

By design, all patients survived and had follow-up documented in the electronic health record as phone contact, clinic or emergency room visits or hospitalizations for at least one month post-surgery. This follow-up generally did not include complete information about retention in a MOUD program. Follow-up was available for at least 6 months in all patients except two in Group I who were lost to follow-up (both at ≥2 months) and two in Group II, both of whom died of non-arrhythmic post-surgical complications, one in the setting of a second episode of endocarditis. As expected, the mean follow-up was longer in Group I (43.2 ± 24.1 months) than in Group II (17.7 ± 9.7 months).

Clinical episodes of cardiac arrest with documented VF occurred in six patients, four in Group I and two in Group II (4/43 IE-CS episodes, 9.3% vs. 2/24 IE-CS episodes, 8.3%, *p* = 0.8914) (Table 3). No episodes of cardiac arrest were associated with monomorphic ventricular tachycardia, pulseless electrical activity or asystole. It is notable that 83% of patients with cardiac arrest had pacemakers. The cardiac arrests occurred at different times during follow-up: one episode occurred during the index hospitalization, three occurred early after discharge and two occurred much later. One Group I patient with a previously placed pacemaker had VF while he was awaiting surgery in the hospital. He was not receiving MOUD at the time, and the arrhythmia was attributed to self-administered opioids. This patient was subsequently treated with buprenorphine and has not had further ventricular arrhythmias documented. Three other patients, all with pacemakers, had cardiac arrest and VF in the first 1–2 months after discharge and in all cases, this was documented to be due to epicardial ventricular lead failure (two patients) or pacemaker management, in the latter case complicated by known asymptomatic non-sustained polymorphic ventricular tachycardia and the need to change her pacing system following a decision to provide sub-cutaneous defibrillator backup. This patient and one other were receiving methadone at the time of their arrests. Two other patients, one in each group, had cardiac arrest significantly later (7 months and 30 months after IE-CS). One patient was taking buprenorphine; the other had missed appointments for methadone-based MOUD and evidence on chart review strongly suggested that a return to opioid use was associated with both arrests.

Five patients already had pacemakers at the time of an index hospitalization (Table 1), and overall, 19 patients were discharged with pacemakers, of whom 5 had transvenous systems (none of whom had lead dysfunction) and 14 had epicardial leads placed at the time of valve surgery. In all cases, the indication for pacing was high-grade or complete heart block, and all but one patient was dependent. Although the numbers are small, the five patients with cardiac arrests did have moderately longer cQTcs (QTc measurements adjusted for their paced QRS) while in the hospital than those with pacemakers but without clinical ventricular arrhythmias (Table 3). No patient was felt to have an indication for a defibrillator prior to pacemaker implantation.

## 4. Discussion

This study had several major findings: 1. With MEET initiation, hospitalized patients with OUD and IE undergoing IE-CS were significantly more likely than a historical group to both receive MOUD during their IE-CS hospitalization, and to be discharged on MOUD. 2. Patients in both groups tended to have moderately prolonged QTc/cQTcs, and 17% had QTcs or cQTcs ≥ 500 ms. This occurred in the setting of the stress of acute illness, surgery and multiple other QT-prolonging factors. 3. The incidence of cardiac arrest due to VF in in both groups was similar, and although low, it was not inconsequential. The majority of these episodes occurred in pacemaker-dependent patients and half were either related to pacemaker epicardial lead malfunction in the early post-discharge period or pacemaker management. Based on a clinical review, late arrhythmic deaths were more likely to be associated with a scenario consistent with a return to opioid use.

It is well recognized that the ongoing opioid epidemic has resulted in a secondary increase in injection drug use associated with infectious complications in general and IE in particular [2,3]. Patients undergoing IE-CS related to injection drug use tend to be younger, have fewer medical co-morbidities, and a lower operative mortality than those without this history. Their midterm survival is similar to that of these other older patients with more medical comorbidities, largely because of a return to opioid use leading to recurrent endocarditis or opioid overdose [14]. Although the perioperative period for patients undergoing IE-CS may be an especially fertile time to engage patients in MOUD, this opportunity is frequently missed. In a recent study, only 24% of patients hospitalized with OUD and IE received an addiction medicine consultation while in the hospital and OUD was mentioned in only 56% of discharge summaries [15]. In this setting, there has been a call for the increased involvement of addiction medicine, other supportive services, and the introduction of MOUD during the IE-CS hospitalization [3,4]. Although inpatient initiation of MOUD has been associated with increased adherence to indicated therapy in general [16], to date, data on longer-term outcomes are conflicting [17,18,19].

The two drugs most often initiated for MOUD in the hospital have been methadone and buprenorphine [16]. Methadone is effective and improves overall survival in patients with OUD [20] but can be associated with QTc prolongation and the risk of ventricular arrhythmias (VF/torsades de pointes). Risk factors for methadone-related QTc prolongation include female gender, longer initial QTc, cocaine use, hepatitis C infection and liver dysfunction, hypokalemia, hypomagnesemia and concomitant use of other QT-prolonging drugs [9,21]. During the years 1969–2002, 5503 adverse events related to methadone were reported to the FDA MedWatch program, including 0.29% related to QT prolongation and 0.78% related to torsades de pointes [6].

In one study, methadone treatment was associated with QT prolongation, defined as a QTc ≥ 470 ms (men) or ≥ 480 ms (women) and/or an increase of ≥ 60 ms compared with the pre-methadone value in 15% of patients [9]. In a French study of patients receiving methadone-based MOUD for an average of 3 years, approximately 10% did have QTc prolongation, none to a value over 500 ms and a review of the French pharmacovigilance system revealed that approximately 3% of reported episodes of torsades de pointes were related to methadone, often in association with therapy initiation or starting other QTc prolonging drugs [22]. QTc values > 450 and < 500 ms were seen in 41–56% of patients and 4–10% had at least one reading > 500 ms for 3 years of follow-up in a US clinic [8]. It is notable that with routine care and the additional stresses of surgery and polypharmacy, the number of our patients with QTcs or cQTcs ≥ 500 ms in both Groups I and II was somewhat higher over the short-term than in most of these reports of chronic therapy and that, at least among pacemaker patients, those who had cardiac arrests due to VF had somewhat longer cQTcs than those who did not, though the numbers are small limiting statistical evaluation (Table 3). This is an indication for a more active management strategy in pacemaker-dependent patients and greater vigilance from cardiology/electrophysiology, as well as careful individual balancing of the positive effects of methadone-based MOUD against these arrhythmic risks to optimize outcomes.

Buprenorphine, which is less likely to significantly increase QTc and carries a lower risk of torsades de pointes [23] has, until recently, been less commonly used in the perioperative setting because of patient choice, a lack of staff familiarity and concern that its pharmacokinetics might interfere with the analgesic efficacy of full mu-opioid receptor agonists used for pain control or precipitate withdrawal [5,23]. Recent data on the success of low-dose buprenorphine initiation in this setting, however, is facilitating greater usage of this safer drug [24].

In both Groups I and II, episodes of cardiac arrest due to VF were similar but not rare (Group I: 9.3% vs. Group II: 8.3% of episodes of IE-CS, *p* = 0.8914) and were especially common in patients with pacemakers. All but one episode occurred after hospital discharge, making comparisons with other patients undergoing valve surgery and requiring pacemakers somewhat more challenging. A cluster of events occurred approximately 1–2 months post-operation, and in all three cases they were associated with either right ventricular epicardial lead failure (two patients) or pacemaker management (one patient). Epicardial pacing systems, frequently placed in this setting because of a desire to either limit intravascular foreign bodies in patients undergoing IE-CS or avoid placing a lead across a prosthetic tricuspid valve, have tended to be less reliable than endocardial pacemaker leads [25], though this has improved with the use of steroid-eluting systems. Pacing thresholds frequently rise early after an implant with both epicardial and endocardial leads [26] and the timing of lead failure at 1–2 months in our patients is consistent with this, especially as our patients did not receive steroid-eluting leads. Loss of ventricular capture in this setting, even if intermittent, would result in bradycardia, which, in itself, prolongs the QTc and causes varying ventricular depolarization and R-R intervals, additional electrical inhomogeneity, possible over- or under-sensing and, especially in the setting of methadone, the potential facilitation of ventricular arrhythmia.

None of our patients had critical ventricular arrhythmias prior to pacemaker implantation, and none were initially discharged with a defibrillator, although one patient was in the process of being upgraded to a defibrillator at the time of her arrest. While sub-cutaneous defibrillators avoid the need for intravascular systems, they do not currently provide pacing support, a confounding issue in these pacemaker-dependent patients. This patient had previously had asymptomatic non-sustained polymorphic ventricular tachycardia while on methadone, resulting in a decrease in her dose and a plan to upgrade her to a subcutaneous defibrillator. Due to pocket complications related to coordinating her initial epicardial pacing system and a subcutaneous defibrillator, she underwent the placement of a VVI leadless pacemaker and immediate defibrillator implantation was deferred. Base pacing was programmed at a low rate to minimize atrio-ventricular dyssynchrony and may have enhanced her previously recognized higher risk for arrhythmia.

### Limitations

This is a single-center, retrospective comparison study, and although long-term follow-up is available for the majority of patients, this is not detailed and does not include information about continued engagement with MOUD. The sample size is relatively small, but this facilitated a very detailed evaluation of the hospital course. We only included patients for whom we had information for ≥ 1 month post-surgery. While it is possible that this enriched our study population with lower-risk patients, we felt the availability of longer-term data was important to our conclusions. During the time period that Group II patients were seen, there was a greater availability of methadone therapy in the community, which may have also impacted our findings. Similarly, experience with buprenorphine use in the perioperative setting has been increasing.

## 5. Conclusions

We found that initiation of a MEET significantly enhanced the use of MOUD in general, and methadone in particular, in patients with OUD during hospitalization for IE-CS. Both Groups I and II had similar moderate and, not infrequently, severe QT prolongation. Episodes of cardiac arrest due to VF were not rare but were similar in both groups, with a cluster being related to pacemaker malfunction/management. Other episodes were believed to be related to the return to opioid use. These events emphasize the need, not only for in-hospital involvement of a MEET, but the importance of its several components, including not just addiction medicine but—especially in pacemaker patients-cardiology/electrophysiology—as well as intensive follow-up during the transition to outpatient management and over the long term for all patients. Further investigation is warranted with regard to both the use of buprenorphine in this setting and the long-term outcome of patients for whom MOUD is initiated during an IE-CS-related hospitalization.

## Figures and Tables

**Table 1:** Characteristics of Groups I and II.

	Group I	Group II	*p*
	n = 38 Unique Patients	n = 24 Unique Patients	
Age, Yrs (median, IQR)	33.5 (29.25–49)	33.5 (25–43)	0.699
Gender, Female n, (%)	8 (21.1)	10 (41.7)	0.084
Reduced EF n, (%)	1 (2.6)	6 (25)	0.007
Hepatitis C n, (%)	26 (68.4)	16 (66.7)	0.890
HIV n, (%)	3 (7.9)	1 (4.2)	0.567
	Group I	Group II	*p*
	n = 43 IE-CS episodes	n = 24 IE-CS episodes	
Pre-surgery			
Paced Rhythm n, (%)	3 (6.9)	2 (8.3)	1
Methadone n, (%)	16 (37.2)	18 (75)	0.005
Buprenorphine n, (%)	4 (9.3)	3 (12.5)	0.695
Surgery			
Paced Rhythm n, (%)	5 (11.6)	6 (25)	0.31
Methadone n, (%)	14 (32.6)	20 (83.3)	<0.001
Buprenorphine n, (%)	1 (2.3)	2 (8.3)	0.29
Peri-discharge			
Paced Rhythm n, (%)	11 (25.6)	8 (33.3)	0.576
Methadone n, (%)	18 (41.9)	19 (79.2)	0.005
Buprenorphine n, (%)	0 (0)	4 (16.7)	0.014

Hepatitis C = Hepatitis C antibody positive; HIV = Human immunodeficiency virus; IE-CS = Infective endocarditis related cardiac surgery; Reduced EF = Ejection fraction < 50%; Yrs = Years.

**Table 2:** Methadone doses.

Methadone Dose (mg/day)	Pre-Surgery	Surgery	Peri-Discharge	*p* (within)
Group I (median, IQR)	70 (40–95)	65 (30–90)	85 (30–90)	0.890
Group II (median, IQR)	47.5 (30–75)	47.5 (30–65)	60 (50–90)	0.484
*p* (between)	0.271	0.653	0.984	

IQR = Interquartile range; mg = Milligram.

**Table 3:** Characteristics of patients with and without VF.

	Pts with VF	Pts without VF
n	6	56
Gender, Female, n (%)	4 (66)	14 (25)
Methadone use *, n (%)	2 (33)	34 (61)
Buprenorphine use at D/C, n (%)	0 (0)	4 (7)
Pacemaker issues, n (%)	3 (50)	0 (0)
Corrected QTc:	PM with VF	PM without VF
	n = 5	n = 14
Pre-surgery cQTc, ms (mean)	477	466
Surgery cQTc, ms (mean)	484	448
Peri-discharge cQTc, ms (mean)	451	414

cQTc = QTc corrected for prolonged QRS; D/C = Discharge; ms = Milliseconds; PM = Pacemaker; VF=Ventricular fibrillation; * = Metahdone use either at time of arrest in those with VF or at time of discharge in patients without VF.

## Data Availability

Not applicable.
